# The VP3 protein of duck hepatitis A virus mediates host cell adsorption and apoptosis

**DOI:** 10.1038/s41598-019-53285-0

**Published:** 2019-11-14

**Authors:** Yalan Lai, Ni Zeng, Mingshu Wang, Anchun Cheng, Qiao Yang, Ying Wu, Renyong Jia, Dekang Zhu, XinXin Zhao, Shun Chen, Mafeng Liu, Shaqiu Zhang, Yin Wang, Zhiwen Xu, Zhengli Chen, Ling zhu, Qihui Luo, Yunya Liu, Yanling Yu, Ling Zhang, Juan Huang, Bin Tian, Leichang Pan, Mujeeb Ur Rehman, Xiaoyue Chen

**Affiliations:** 1 Institute of Preventive Veterinary Medicine, Sichuan Agricultural University, Wenjiang, Chengdu City, 611130 Sichuan P.R. China; 2 Key Laboratory of Animal Disease and Human Health of Sichuan Province, Sichuan Agricultural University, Wenjiang, Chengdu City, 611130 Sichuan P.R. China; 3 Avian Disease Research Center, College of Veterinary Medicine, Sichuan Agricultural University, Wenjiang, Chengdu City, 611130 Sichuan P.R. China

**Keywords:** Viral host response, Virus-host interactions

## Abstract

Duck hepatitis A virus (DHAV) causes an infectious disease that mainly affects 1- to 4-week-old ducklings, resulting in considerable loss to the duck industry. Although there have been many studies on DHAV in recent years, the effects on host infection and pathogenesis of DHAV-1 remain largely unknown. This study investigated the effects of the DHAV-1 structural protein VP3 on DHAV-1 virus adsorption and apoptosis to explore the role of VP3 in the viral life cycle. The effects of DHAV-1 VP3 and an antibody against the protein on virion adsorption was analyzed by qRT-PCR. The results showed that the virus copy number for the rabbit anti-VP3 IgG-treated group was significantly lower than that for the negative control group but higher than that for the rabbit anti-DHAV-1 IgG-treated group. This result indicates that VP3 mediates DHAV-1 virus adsorption but that it is not the only protein that involved in this process. In addition, a eukaryotic recombinant plasmid, pCAGGS/VP3, was transfected into duck embryo fibroblasts (DEFs), and the apoptotic rate was determined by DAPI staining, the TUNEL assay and flow cytometry. DAPI staining showed nucleus fragmentation and nuclear edge shifting. TUNEL assay results revealed yellow nuclei, and flow cytometry indicated a significant increase in the apoptotic rate. In addition, qRT-PCR revealed increased in the transcriptional levels of the apoptotic caspase-3, −8 and −9, with the largest increase for caspase-3, followed by caspase-9 and caspase-8. Enzyme activity analysis confirmed these results. Furthermore, the VP3 protein decreased the mitochondrial membrane potential, and the transcriptional levels of the proapoptotic factors Bak, Cyt c and Apaf-1 in the mitochondrial apoptotic pathway were significantly upregulated. These data suggest that expression of VP3 in DEFs induces apoptosis and may primarily activate caspase-3-induced apoptosis through mitochondrion-mediated intrinsic pathways. The findings provide scientific data to clarify DHAV-1 infection and pathogenesis.

## Introduction

Duck viral hepatitis (DVH) causes an infection that mainly affects 1- to 4-week-old ducklings. The main symptoms of this disease are spasm, convulsions, opisthotonos, hepatomegaly and hepatic hemorrhage^1^. DHV was once divided into three serotypes (DHV-I, DHV-II, and DHV-III), with no cross-immunogenicity between them^2–4^. However, DHV-I was later renamed duck hepatitis A virus (DHAV) and assigned to genus *Avihepatovirus* of *Picornaviridae*. DHAV can be further divided into three genotypes^5^ (DHAV-1, DHAV-2^6^ and DHAV-3^7,8^), the most common of which are DHAV-1 and DHAV-3 in China^9^.

DHAV-1 virions are spherical or spheroidal and nonencapsulated, and the viral capsid has an icosahedral structure composed of 12 pentamers, with each pentamer consisting of a VP1-VP4 capsid protein complex. The genome of DHAV-1 is a single positive-strand RNA with the complete genomic structure VPg + 5 ´UTR-[VP0-VP3-VP1/2 A(2A1-2A2-2A3)−2B-2C/3A-3B-3C-3D]-3 ´UTR + Poly(A)^10^. The genome has only one open reading frame (ORF) and can be divided into three regions, including the P1 region, which can be decomposed into genes encoding structural proteins (VP0, VP3, and VP1), and the P2 and P3 regions, which encode viral nonstructural proteins^11,12^.

The mutation hotspot region of picornavirus family genomes is mainly located in the P1 region, and the mutagenicity of the four capsid proteins is from high to low VP1 > VP3 > VP2 > VP4^13,14^. Although the variability of VP3 is lower than that of VP1, the high VP3 variability might result in different functions within the same species. Functions of picornavirus VP3 have been well investigated. For example, VP3 has good immunogenicity^15^ and induces cellular immunity^16–18^. VP3 can also bind to cell surface receptors because its amino acid side chain is exposed on the surface of the viral capsid^19^, and it plays a role in the virus adhesion process during host cell invasion. Additionally, VP3 can induce apoptosis^20^.

In recent years, studies on the pathogenesis^21–25^, diagnosis^26–29^, immunity^23,30^ and partial gene function^31,32^ of DHAV-1 have been reported. Since the first discovery of DVH in 1949^33^, the disease has been reported in various parts of the world. DHAV-1 is one of the most severe genotypes^34,35^; however, knowledge about the VP3 protein of DHAV-1 is still limited. For instance, the effects of DHAV-1 on host infection and pathogenesis are mainly unclear. In particular, does the capsid protein VP3 of DHAV-1 affect the adsorption of DHAV-1 on host cells? How does VP3 affect the adsorption of DHAV-1 on host cells? What protein does VP3 interact with in the host cell? Does the VP3 protein of DHAV-1 induce apoptosis and how does this occur? To provide scientific data that can be used to elucidate the infection and pathogenesis of DHAV-1, this study explored the effects of VP3 on DHAV-1 adsorption and apoptosis induction.

## Results

### Blocking effect of rabbit anti-VP3 IgG on DHAV-1 adsorption

**Figure 1:**
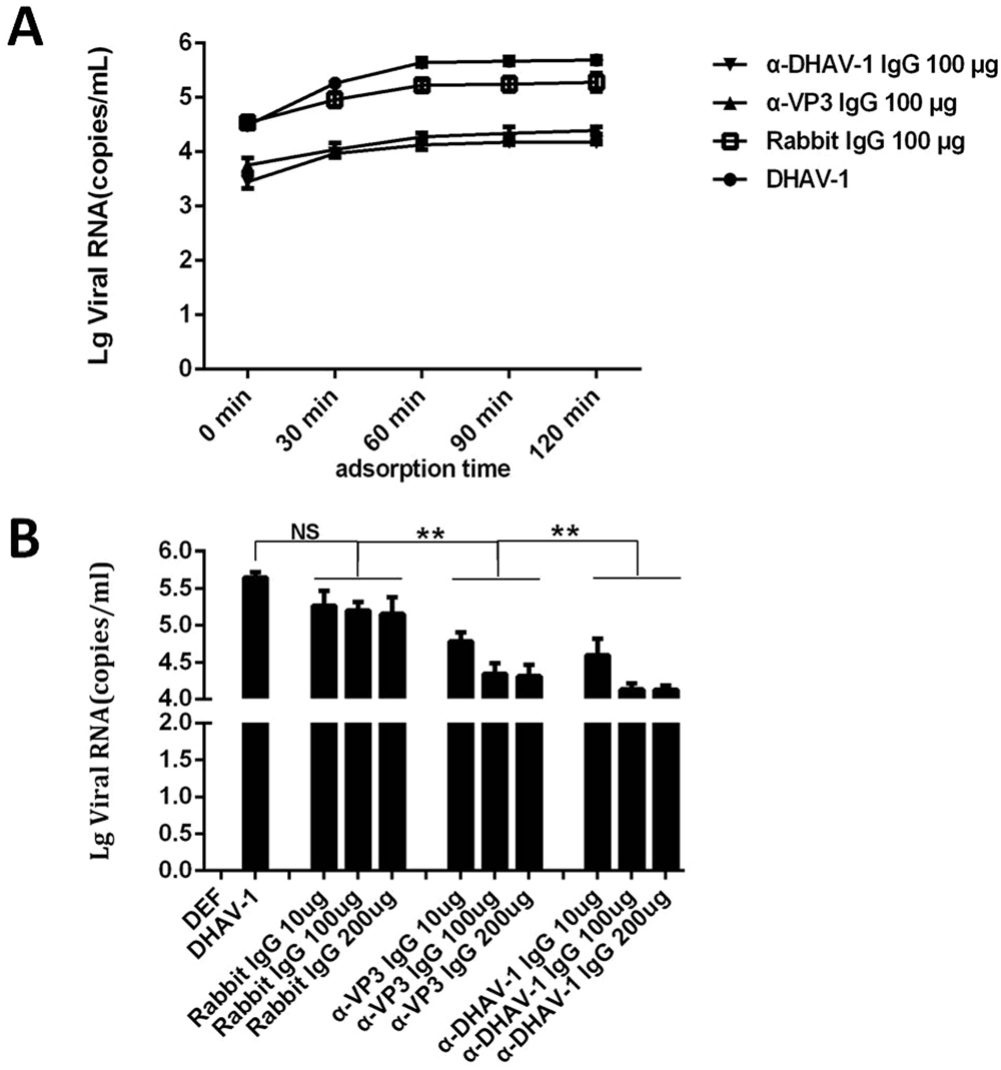
Detection results of blocking inhibition of DHAV-1 by rabbit anti-VP3 IgG and rabbit anti-DHAV-1 IgG. (**A**) Blocking effect of rabbit anti-VP3 IgG (α-VP3 IgG) and rabbit anti-DHAV-1 IgG (α-DHAV-1 IgG) on DHAV-1 adsorption at different times. (**B**) Adsorption inhibition assay of DHAV-1 by different doses of rabbit anti-VP3 IgG and rabbit anti-DHAV-1 IgG.

To investigate the blocking effect of rabbit anti-VP3 IgG (α-VP3 IgG) on DHAV-1 at different times, 100 μg of rabbit anti-VP3 IgG, rabbit anti-DHAV-1 IgG, and normal rabbit IgG was incubated with 200 μL of DHAV-1 (5 × 10^6.6^ copies/μL) at 37 °C for 1 h and then added to a DEF monolayer for adsorption at 4 °C. Cell samples were collected at different times (0, 10, 60, 90, and 120 min), and virus copy number was determined by one-step fluorescence quantitative RT-PCR. The results showed that the amount of virus adsorbed gradually increased with time. Maximum adsorption was reached at 60 min, after which the amount of virion adsorption tended to stabilize (Fig. 1A).

To investigate the ability of different doses of rabbit anti-VP3 IgG on DHAV-1 (200 μL) to block adsorption, we incubated different doses (10, 100 and 200 μg) of purified rabbit anti-VP3 IgG, rabbit anti-DHAV-1 IgG and normal rabbit IgG with 200 μL of DHAV-1 (5 × 106.6 copies/μL) virus at 37 °C for 1 h and then added the sample to the DEF monolayer at 4 °C for 60 min. Cell samples were collected, and virus copy number was determined by one-step fluorescence qRT-PCR. The results showed that the blocking effect was maximized when 100 μg of rabbit anti-VP3 IgG and 100 μg of rabbit anti-DHAV-1 IgG were added (Fig. 1B).

As shown in Fig. 1A,B, there was no significant difference between the amount of DHAV-1 virus adsorbed by the normal rabbit IgG-treated group compared with the untreated group (P > 0.05). Compared with the amount of DHAV-1 virus adsorbed by the normal rabbit IgG (rabbit IgG)-treated group and the untreated group, the virion copy number on the cell surface of rabbit anti-VP3 IgG and rabbit anti-DHAV-1 IgG treatment groups decreased significantly (P < 0.05). However, when the amount of virus adsorption and antibody blocking were optimal, the viral copy number of the rabbit anti-VP3 IgG-treated group was still higher than that of the rabbit anti-DHAV-1 IgG-treated group (Fig. 1).

### Blocking effect of VP3 on DHAV-1 adsorption

To further investigate the blocking effect of the VP3 protein on DHAV-1 adsorption, different doses (10, 100 and 200 μg) of GST-VP3 fusion protein, GST-tagged protein and BSA were added to DEFs for 1 h at 4 °C. The protein-treated DEFs were incubated with 200 μL of DHAV-1 (5 × 106.6 copies/μL) at 4 °C for 1 h (to allow virus adsorption) and then at 37 °C for 2 min (to allow virus to permeate). Cell samples were then collected and analyzed by one-step fluorescence qRT-PCR.

**Figure 2:**
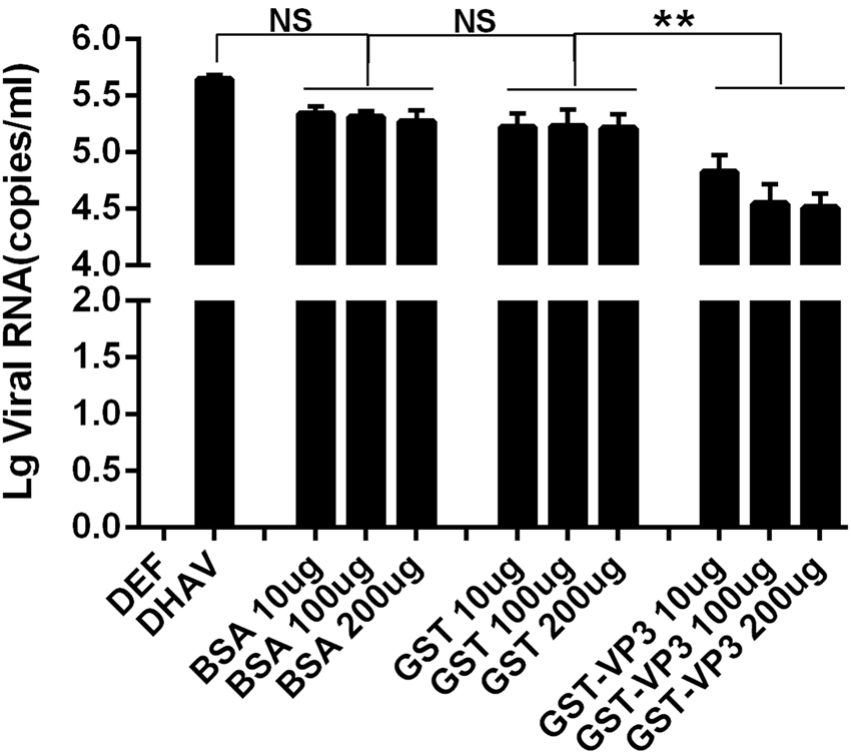
Detection results of blocking DHAV-1 adsorption by different doses of VP3 protein.

According to the results, there was no significant difference in the amount of DHAV-1 virus adsorbed the presence of GST soluble label protein (GST) and BSA (BSA) and that of untreated DHAV-1 (DHAV-1) virus. However, the number of viral copies on the cell surface of the GST-VP3 soluble fusion protein (GST-VP3) group was significantly reduced (P < 0.05), and the competition blocking effect was maximized when 100 μg of GST-VP3 was added. Under these conditions, adsorption of the virus was still not completely inhibited (Fig. 2).

### Construction, identification and expression of eukaryotic recombinant plasmid pCAGGS/VP3

**Figure 3:**
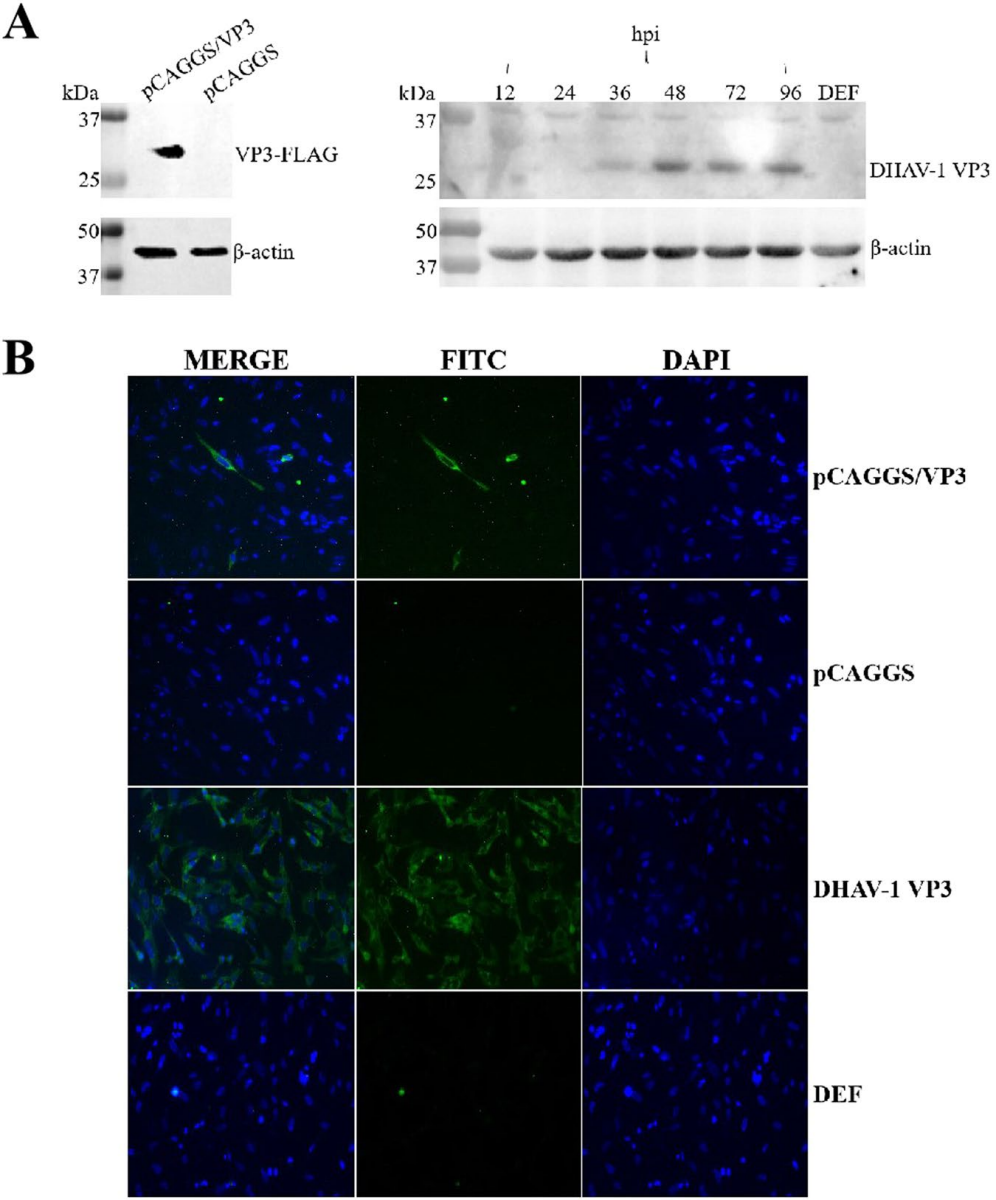
Expression and identification of eukaryotic recombinant plasmid pCAGGS/VP3. In this experiment, β-actin was selected as an internal reference. In addition, complete blots are presented in Supplementary Figs 11 and 12. (**A**) Expression of VP3 after transfection of pCAGGS/VP3 into DEF and expression of VP3 after DHAV-1 infection with DEF. (**B**) Localization of VP3 in pFAGGS/VP3-transfected and DHAV-1-infected DEFs. IFA detection of VP3 in DEFs after transfection of pCAGGS/VP3 and pCAGGS for 48 h and IFA detection of VP3 after DEFs were infected or not with DHAV-1 for 48 h were performed.

To explore the role of the VP3 protein in inducing apoptosis, we first constructed the eukaryotic recombinant plasmid pCAGGS/VP3 and examined its expression in DEFs as well as that of the DHAV-1 VP3 protein during virus proliferation by Western blotting. The results indicated a specific band of the expected size of approximately 28 kDa, with no specific band in the negative control. VP3 protein expression increased with time in DEFs after DHAV-1 infection, reached a maximum at 48 h, and then began to decrease. Comparison of the above two results showed no significant difference in size or immunogenicity between the transfected overexpressed protein and the protein expressed during viral infection (Fig. 3A). Expression of VP3 after transfection of pCAGGS/VP3 into DEFs for 48 h and after DHAV-1 infection with DEFs for 48 h was also detected by an indirect immunofluorescence technique. The VP3-FLAG protein expressed in the overexpression group and the VP3 protein expressed in the infected group (Fig. 3B) were distributed in the cytoplasm, with no significant difference between the two with regard to subcellular localization.

### Detection of apoptosis in DEFs induced by VP3 protein

**Figure 4:**
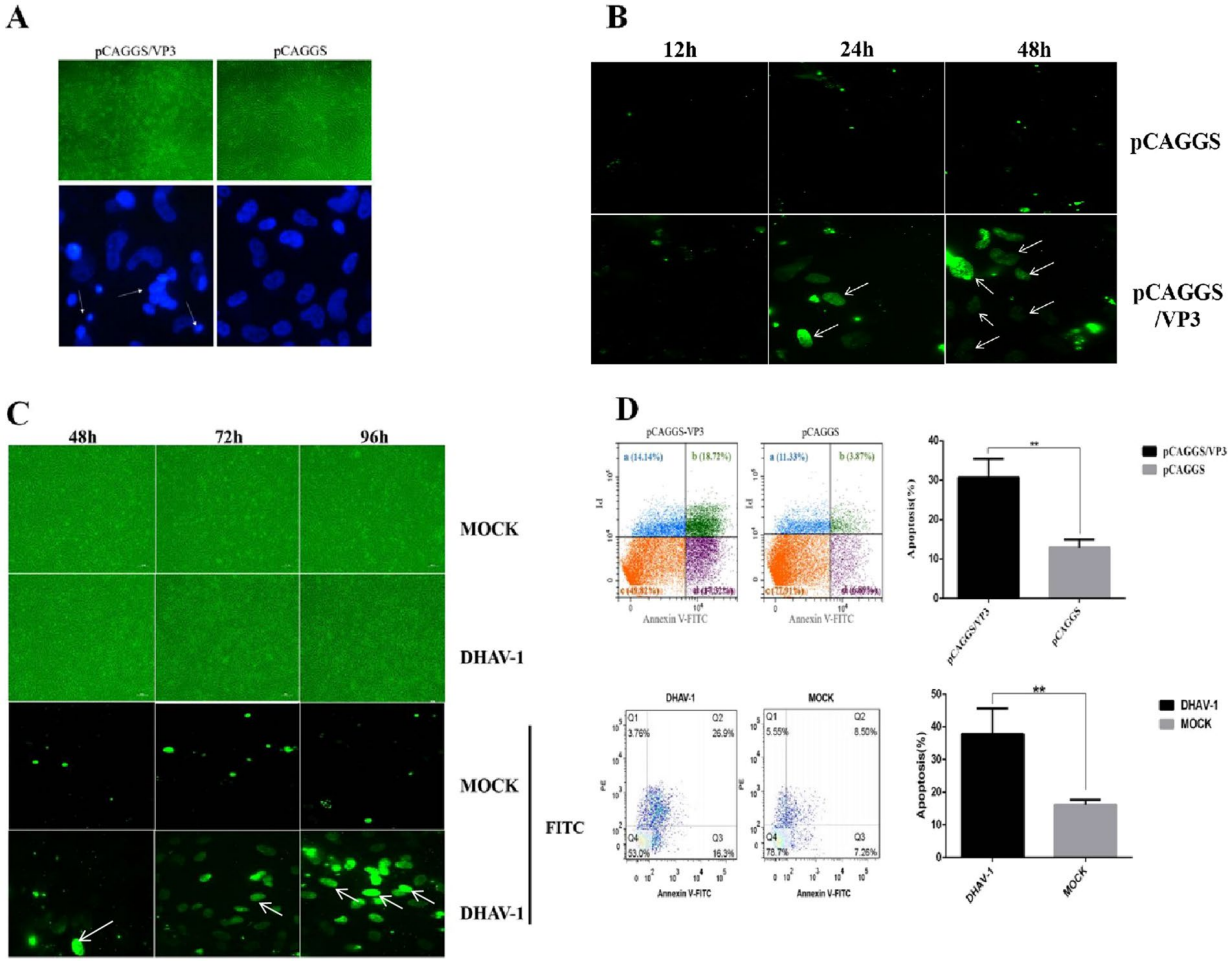
Detection of VP3 protein-induced apoptosis in DEF cells. (**A**) Cell morphology and nuclear DAPI staining of DEFs transfected with pCAGGS/VP3 for 48 h. (**B**) TUNEL staining of pCAGGS/VP3-transfected DEFs. DEFs were transfected with pCAGGS/VP3 or pCAGGS for 48 h and observed under a 60× objective lens. Apoptotic cells are shown by the arrows. (**C**) TUNEL staining of DEFs infected with DHAV-1. DEFs were infected with DHAV-1 for 48 h, 72 h, and 96 h and observed under a 60× objective lens. (**D**) Flow cytometry results of pFAGGS/VP3-transfected DEFs and DHAV-1-infected DEFs. Scatter plot based on flow cytometry of apoptotic DEFs after transfection with pCAGGS/VP3 or pCAGGS for 48 h or after DHAV-1 infection for 96 h (a, b, c, and d are dead cells, early apoptotic cells, viable cells, and late apoptotic cells, respectively) and data analysis of the proportion of total apoptotic cells (sum of the ratio of cells in the b and d regions).

Compared with pCAGGS transfection, transfection of pCAGGS/VP3 into DEFs for 48 h resulted in clearer cell fragmentation, morphology of greater irregularity, and nuclear fragmentation and obvious nuclear lateral migration (Fig. 4A). A one-step TUNEL Apoptosis Detection Kit (Beyotime Biotechnology) was used to perform TUNEL staining on pCAGGS/VP3-transfected DEFs, whereby cells undergoing apoptosis will emit green fluorescence compared with the pCAGGS group; the cells were stained with Annexin V-FITC/PI dye and detected by flow cytometry. Distinct apoptotic cells were observed for the pCAGGS/VP3 group (Fig. 4B). As shown in Fig. 4D, apoptotic cells (including early apoptotic cells and late apoptotic cells) in the three pCAGGS/VP3-transfected groups accounted for 36.64%, 29.06%, and 26.92% of the total cells, respectively. The apoptosis rates of the three pCAGGS-transfected groups were 10.76%, 13.03%, and 14.90%, respectively, and the difference was significant (P < 0.05). The above results indicate that VP3 protein expression can induce apoptosis in DEFs. At the same time, DEFs were infected with DHAV-1, and we observed no obvious apoptosis in the DHAV-1 infection group compared with the MOCK group at 12 h-36 h after infection. Therefore, we used TUNEL and flow cytometry to detect apoptosis in the DHAV-1-infected group from 48 h to 96 h after infection. The results are shown in Fig. 4C,D. The level of apoptosis in the DHAV-1 infected group was significantly higher than that in the MOCK group, indicating that DHAV-1 can induce DEF apoptosis.

### Detection of apoptotic caspase-3, −8 and −9 transcriptional levels and enzyme activity after transfection of pCAGGS/VP3 into DEFs

**Figure 5:**
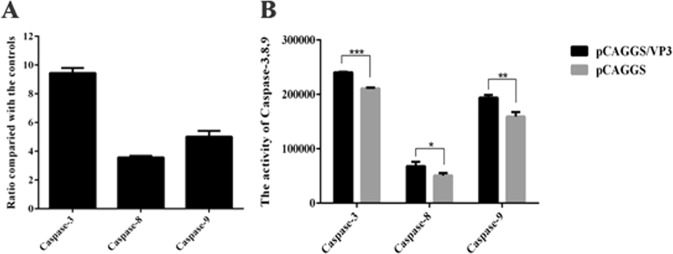
Detection results of apoptotic caspase-3, −8 and −9 transcriptional levels and enzyme activities. (**A**) Detection of transcript levels of apoptotic caspase-3, −8 and −9 in pCAGGS/VP3-transfected DEFs. (**B**) Detection of enzymatic activity of apoptotic caspase-3, −8 and −9 in pCAGGS/VP3-transfected DEFs.

After DEFs were transfected with pCAGGS/VP3 and pCAGGS for 48 h, qRT-PCR showed that detect caspase-3, −8 and −9 transcription was upregulated. Specifically, VP3-FLAG overexpression upregulated caspase-3 transcription by approximately 9-fold, that of caspase-8 by approximately 3-fold, and that of caspase-9 by approximately 5-fold (Fig. 5A). Additionally, the enzyme activities of caspase-3, caspase-8 and caspase-9 determined using kits were compared between the pCAGGS/VP3 and pCAGGS transfection groups. Overall, there was a significant increase in activitt (0.01 < P < 0.05 for *, P < 0.01 for **), with caspase-3 having the highest increase, followed by caspase-9, and caspase-8 activity (Fig. 5B), consistent with the results presented in Fig. 5A.

### Effect of VP3 on mitochondrial membrane potential

**Figure 6:**
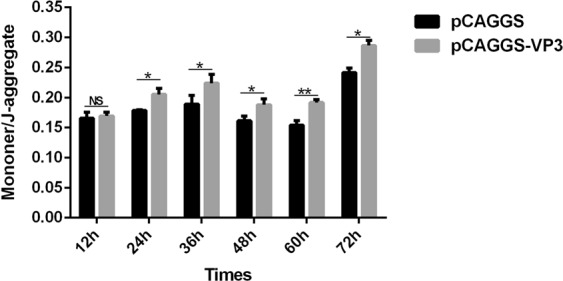
Determination of mitochondrial membrane potential (MMP) in DEFs. A multi-function microplate reader was used to evaluate the mitochondrial membrane potential of DEFs. Data are expressed as the mean ± SD of three independent experiments. *p < 0.05 and **p < 0.01, compared with the control group.

Because pCAGGS/VP3 transfection of DEFs can upregulate the transcriptional level and activity of caspase-9, which is mainly responsible for the mitochondrial apoptotic pathway, we suspected that VP3 induces apoptosis mainly through the mitochondrial pathway. As decreased mitochondrial membrane potential (MMP) is one of the hallmarks of mitochondrial apoptotic pathway activation, we examined the effect of VP3 protein on mitochondrial membrane potential using the mitochondrial JC-1 probe. The mitochondrial membrane potential in normal cells is high, and JC-1 is concentrated in the mitochondrial matrix, emitting red fluorescence; when the mitochondrial membrane potential is decreased, JC-1 enters the cytoplasm in a monomeric form and emits green fluorescence. The results of this assay are shown in Fig. 6. At 24 h-72 after transfection, the ratio of JC-1 monomer to aggregate in cells transfected with pCAGGS/VP3 was significantly higher than that in cell transfected with pCAGGS. The difference was most obvious at 60 h after transfection.

### Effect of VP3 on the transcriptional level of apoptotic factors

**Figure 7:**
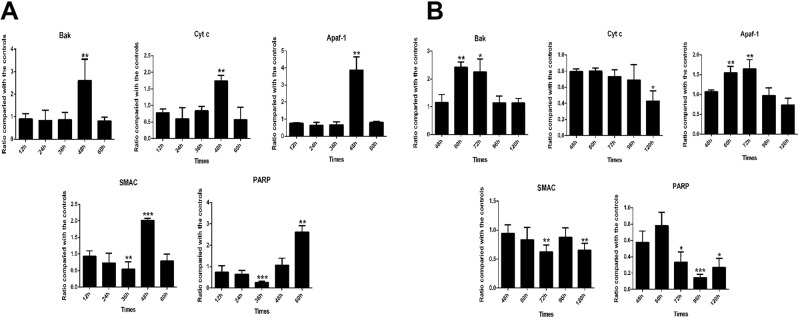
Detection of transcriptional levels of apoptotic proteins of the mitochondrial pathway. (**A**) Detection of transcriptional levels in pCAGGS/VP3-transfected DEFs. (**B**) Detection of transcriptional levels in DHAV-1-infected DEFs. *p < 0.05, **p < 0.01, ***p < 0.001, compared with the control group.

We subsequently examined the transcriptional levels of the proapoptotic factors Bak, Cyt c, and Apaf-1 in the mitochondrial apoptotic pathway after pCAGGS/VP3 transfection. As depicted in Fig. 7A, the levels of Bak, Cyt c and Apaf-1 in the pCAGGS/VP3 group were upregulated by approximately 2.5-fold, 1.5-fold and 3.5-fold, respectively, at 48 h after transfection compared with the pCAGGS group. Bak and Apaf-1 in the DHAV-1 infection group increased significantly at 60 h and 72 h after infection, whereas Cyt c was downregulated at 120 h after infection (Fig. 7B).

**Figure 8:**
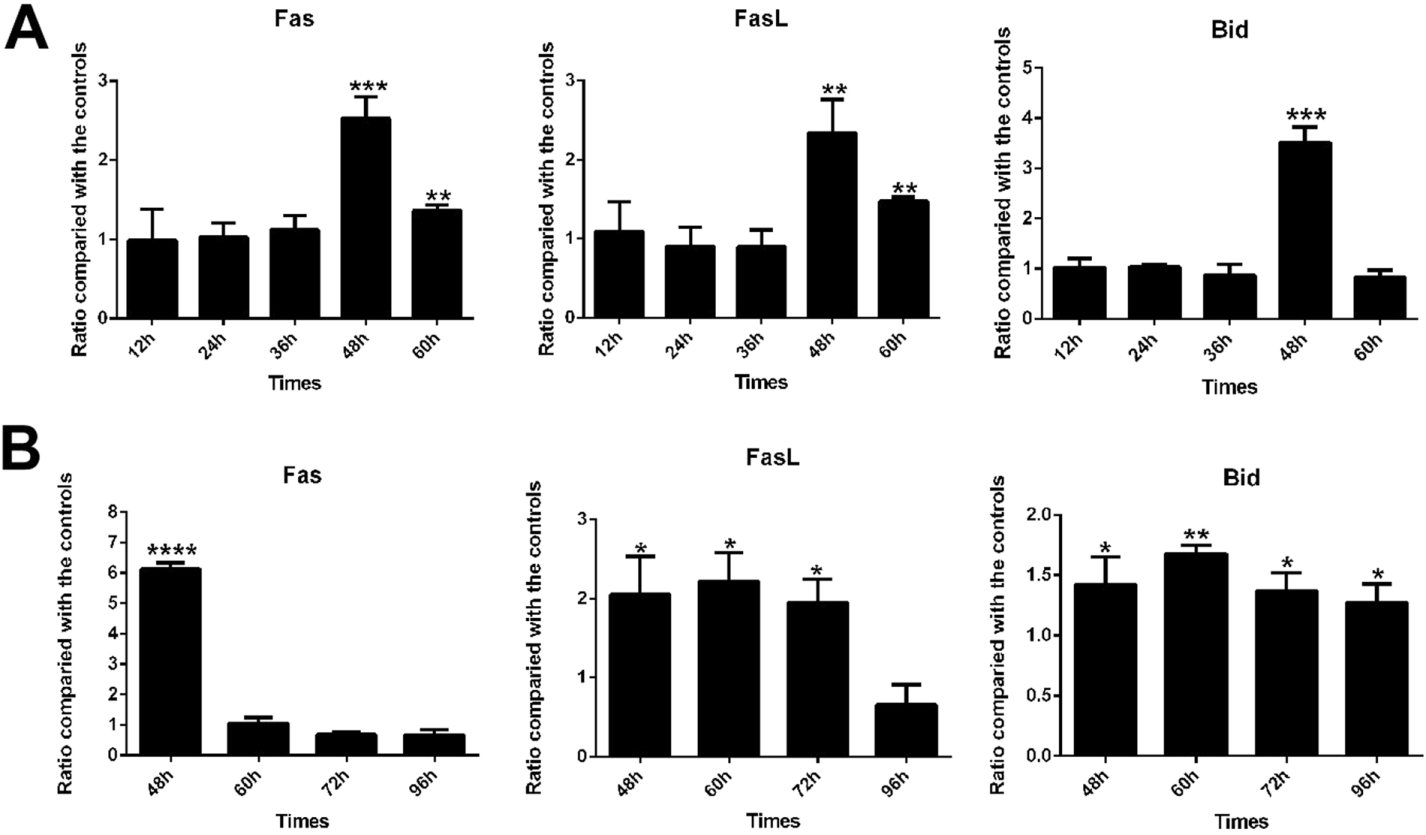
Detection of transcriptional levels of apoptotic proteins of the death receptor-mediated apoptosis pathway. (**A**) Detection of transcriptional levels in pCAGGS/VP3-transfected DEFs. (**B**) Detection of transcriptional levels in DHAV-1-infected DEFs. *p < 0.05, **p < 0.01, ****p < 0.0001, compared with the control group.

Caspase-8 is mainly responsible for the death receptor-mediated apoptosis pathway, and the VP3 protein upregulated its transcriptional level and enzyme activity. We also examined the proapoptotic factors Fas, FasL and Bid in the death receptor-mediated apoptotic pathway, and the results are shown in Fig. 8A. At 48 h after transfection, the transcriptional levels of Fas, FasL, and Bid were upregulated by approximately 2.5-fold, 2.5-fold, and 3.5-fold, respectively, in the pCAGGS/VP3 group compared with the transfected pCAGGS group. In addition, Fas in the DHAV-1 infection group was significantly upregulated at 48 h after infection, and FasL and Bid were significantly upregulated from 48 h to 72 h after infection (Fig. 8B).

### Effect of VP3 on the PI3K/AKT signaling pathway

**Figure 9:**
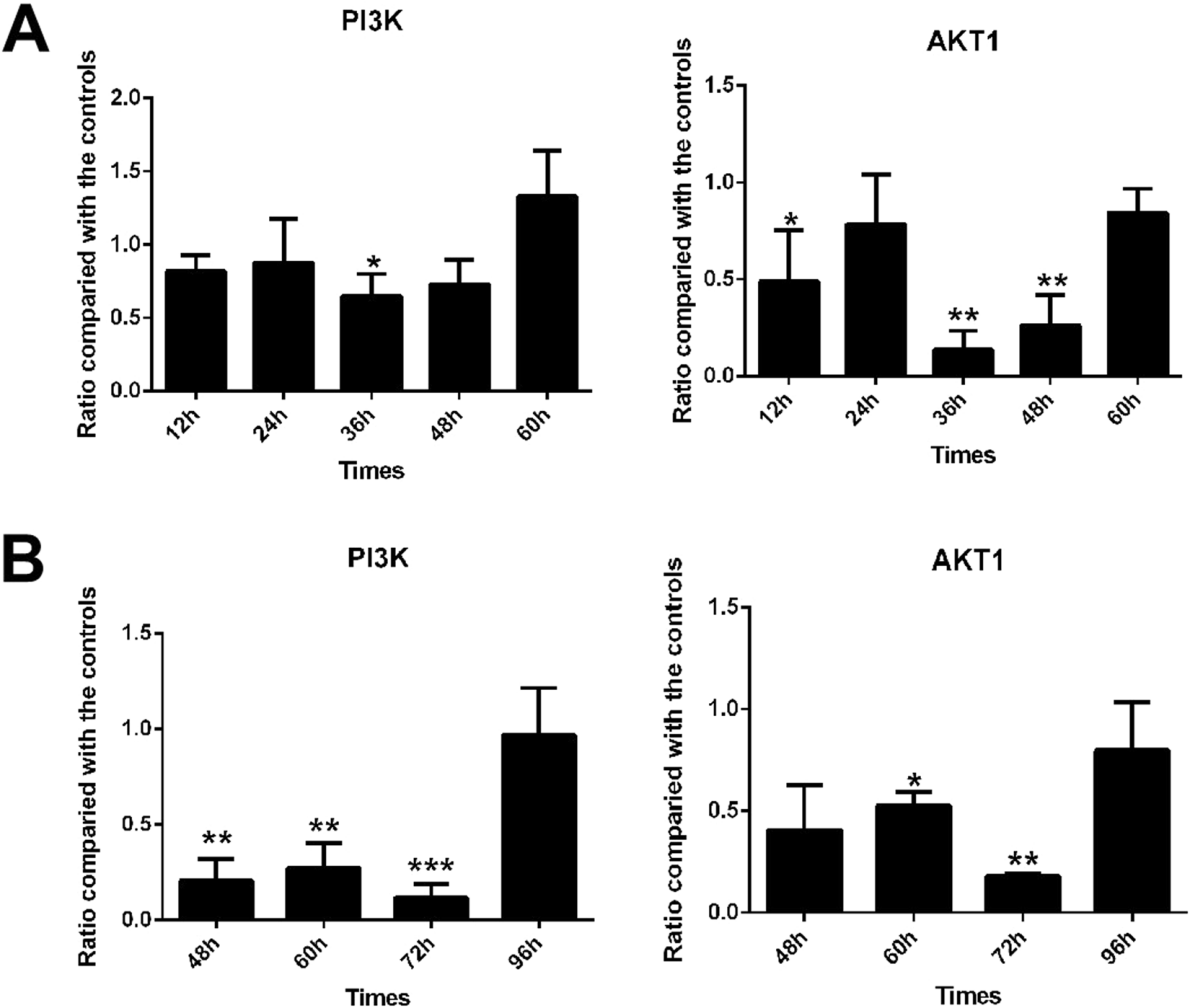
Results of transcript level detection of PI3K and AKT1 of the PI3K/AKT survival pathway. (**A**) Detection of transcriptional levels in pCAGGS/VP3-transfected DEFs. (**B**) Detection of transcriptional levels in DHAV-1-infected DEFs. *p < 0.05, **p < 0.01, ***p < 0.001, compared with the control group.

The PI3K/Akt survival pathway is an important anti-apoptotic signaling pathway composed mainly of 3-phosphoinositide kinase (PI3K) and protein kinase B (Akt). Therefore, we examined the effect of VP3 on the transcriptional levels of PI3K and AKT. Compared with the pCAGGS group, PI3K in the pCAGGS/VP3 group was significantly downregulated at 36 h after transfection, and AKT1 was significantly downregulated 36 h–48 h after transfection (Fig. 9A). PI3K in the DHAV-1-infected group was downregulated from 48 h to 72 h after infection and AKT from 60 h to 72 h after infection (Fig. 9B).

## Discussion

Similar to most viruses, the first stage of picornavirus infection in susceptible cells is mediated by interaction between the viral capsid with specific receptors on the cell membrane. This phase of attachment is also a measure of tissue tropism. Therefore, adsorption of the virus on the host cell is an important part of viral invasion^36,37^. Although there have been a few studies on the VP3 protein of picornaviruses, there are scarce studies on adsorption. For example, Smyth S M *et al*. reported that five residues of the VP3 protein of bovine enterovirus (BEV) form a cyclic structure associated with receptor binding^37^. In addition, FMDV binds to the cell surface heparan sulfate (HS) receptor independently of the integrin receptor^38^, and the 85th amino acid of FMDV VP3 is involved in the formation of the bottom of the HS receptor binding pocket^39^. The 56th residue of VP3 also plays an important role in this recognition^40,41^. Our previous studies confirmed that DHAV-1 VP3 amino acids 1–20, 131–150 and 200–209 are linear B cell epitopes and that rabbit anti-VP3 serum has good neutralizing activity against DHAV-1^16^. In the present study, the amount of DHAV-1 virus adsorption of the samples treated with different doses of rabbit anti-VP3 IgG was significantly reduced compared with that of the untreated group. However, virus adsorption was not completely inhibited, which indicates that rabbit anti-VP3 IgG has a blocking effect on DHAV-1 adsorption but does not completely block it on DEFs. Li *et al*.^42^ reported that the VP1 protein of DHAV-1 is a target of neutralizing antibodies^42^, and our experimental results are in agreement.

However, if a protein binds to a receptor on the cell surface and that site is a receptor for the virus, the protein will inhibit viral infection of a susceptible cell by occupying the binding site. In this study, we treated DEFs with different doses of GST-VP3 soluble fusion protein and found that the amount of DHAV-1 virus adsorbed was significantly less than that for normal cells, indicating that the GST-VP3 recombinant protein and DHAV-1 use the same cell binding site on the DEF surface. Nonetheless, the specific mechanism of the interaction between VP3 and other proteins and the role of VP3 are still unclear and require further study.

In addition to being involved in viral adsorption, surface structural proteins of many members of the picornavirus family can induce apoptosis in host cells. For example, Peng J M *et al*.^43^ reported that recombinant VP1 of FMDV binds to the integrin of BHK-21 cells *in vitro*, inactivating Akt and activating proapoptotic responses such as glycogen synthase kinase 3β dephosphorylation. Caspase-9 cleavage eventually leads to apoptosis of BHK-21 cells^43^, and Henke A *et al*.^44^ found that the capsid protein VP2 of coxsackievirus B3 (CVB3) induces apoptosis in host cells by specifically interacting with the proapoptotic protein Siva44. Moreover, according to Liu J *et al*. ^20^, VP3 of avian encephalomyelitis virus (AEV) induces apoptosis in Cos-7 and chicken embryonic brain (CEB) cells^20^. In DHAV-1-infected ducklings, cells exhibit obvious apoptosis^22^, but analysis of specific viral proteins related to apoptosis remains limited. Regarding viral protein research of DHAV-1, only protein 2 A has been reported to date to induce apoptosis^45^. In the present study, the pCAGGS/VP3 group showed cell fragmentation, nuclear fragmentation, and nuclear lateral migration compared to the pCAGGS group (Fig. 4A), and these results are consistent with the main features of apoptosis^44^. Subsequently, we transfected pCAGGS/VP3 into DEFs and performed TUNEL staining and flow cytometry, revealing significant rates of apoptosis (Fig. 4B,C), which indicates that VP3 can indeed induce apoptosis in DEFs. At the same time, DHAV-1 also induced apoptosis in DEFs (Fig. 4C,D), indicating that VP3 is an important player in DHAV-1-induced apoptosis.

Morphological and nuclear changes associated with apoptosis are usually caused by sequential activation of the caspase family, and caspase-3 is the most important apoptosis-inducing enzyme in this family^46,47^. Apoptosis is mainly accomplished via two pathways: one involving caspase-8 stimulation by activating cell surface death receptors to trigger the exogenous apoptosis pathway (death receptor-mediated apoptosis pathway); and another involving caspase-9 stimulation via release of proapoptotic factors from mitochondria to trigger the internal apoptotic pathway. Regardless, both pathways ultimately cause apoptosis by activating caspase-3. In addition, these two pathways can be linked by Bid in the Bcl-2 family^47^. After transfection of pCAGGS/VP3 into DEFs, the transcriptional levels of apoptotic caspase-3, −8 and −9 were upregulated. Overexpression of VP3-FLAG in DEFs increased the transcriptional levels of apoptotic caspase-3, −8 and −9, with the largest increase in caspase-3, followed by caspase-9 and caspase-8, and enzyme activity tests confirmed these results (Fig. 5). We also examined the mitochondrial membrane potential of DEFs and found that of the pCAGGS/VP3-transfected group to be significantly lower than that of the pCAGGS-transfected group (Fig. 6). Decreased mitochondrial membrane potential is one of the hallmarks of activation of the mitochondrial apoptotic pathway, which suggests that VP3 may activate caspase-3 in DEFs to induce apoptosis mainly through the mitochondrion pathway and that the death receptor-mediated apoptotic pathway may play a secondary role.

We then examined the transcriptional levels of various apoptotic factors in the mitochondrial apoptotic pathway. At 48 h after transfection, the levels of proapoptotic factors Bak, Cyt c and Apaf-1 in the pCAGGS/VP3 group were significantly upregulated. Bak can be activated by the formation of heterodimers or homodimers, resulting in a decrease in mitochondrial membrane potential and release of Cyt c^48^. Cyt c activates caspase-9 by forming a complex with pro-caspase-9 and the apoptotic protease activator Apaf-1, thereby activating caspase-3^49^. The findings suggest that VP3 may downregulate Bak and decrease the mitochondrial membrane potential, which triggers the mitochondrial apoptosis pathway. At the same time, VP3 promotes the mitochondrial apoptosis pathway by upregulating Cyt c and Apaf-1 and ultimately induces apoptosis. The transcriptional levels of Bak, Cyt c, Apaf-1 and other factors were not upregulated after 60 h of transfection, which may have been due to decreased expression of VP3 or massive cell death. However, Cyt c was downregulated at 120 h after infection in the DHAV-1 infection group, which may be related to the effects of other viral proteins; the specific mechanism remains to be studied.

We subsequently examined the transcriptional levels of various apoptotic factors in the death receptor-mediated apoptotic pathway in the pCAGGS/VP3 group and observed Fas, FasL and Bid to be significantly upregulated at 48 h after transfection. Binding of the death ligand FasL to its corresponding receptor Fas triggers the death receptor-mediated apoptotic pathway, suggesting that VP3 may function to regulate the this pathway, possibly by upregulating Fas and FasL to enhance caspase-8 activation; VP3 also upregulates Bid to enhance the mitochondrial apoptotic pathway. As the PI3K/Akt survival pathway is an important anti-apoptotic signaling pathway *in vivo*, and we explored the effect of VP3 on this pathway. The results indicated that VP3 has an inhibitory effect on PI3K and AKT1 (Fig. 9A); thus, VP3 may inhibit the PI3K/AKT survival pathway to promote apoptosis. In summary, the VP3 protein may induce apoptosis through multiple pathways, promoting the mitochondrial and death receptor-mediated apoptosis pathways and inhibiting the PI3K/AKT survival pathway. However, due to the lack of duck-sourced antibodies and ELISA kits, the effect of VP3 on the protein levels of various apoptotic factors is unknown, and further research is needed.

**Figure 10:**
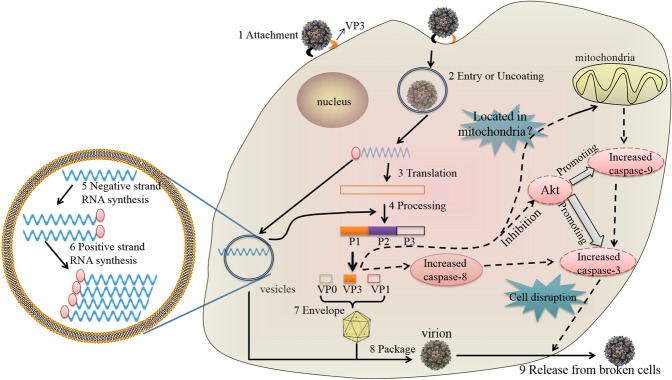
Overview of the picornavirus replication cycle. Steps 1–9 are the replication cycle of picornaviruses^52–54^. The dotted line indicates the pathway by which the VP3 protein induces apoptosis^47^.

Based on the above results, the structural protein VP3 plays an important role in the disease cycle of the virus. To explore the role of the DHAV-1 VP3 protein in the viral life cycle, we selected the beginning (adsorption phase) and end (virus release) of the life cycle as entry points^19,20^ to further elucidate the function of VP3 (Fig. 10). The results demonstrated that VP3 can indeed contribute to the adsorption of DHAV-1 and also induce apoptosis in host cells. Overall, we confirmed that DHAV-1 2 A and VP3 can induce apoptosis, though there are few studies on the inhibition of apoptosis. Therefore, many aspects of DHAV-1 remain to be explored.

## Materials and Methods

### Ethics statement

All duck embryo experiments were approved by the Committee of Experiment Operational Guidelines and Animal Welfare of Sichuan Agricultural University, with approval number 2018102030-1807. Experiments were conducted in accordance with approved guidelines.

### Strain and antibody

The DHAV-1 H strain (GenBank accession number: JQ301467.1), rabbit anti-VP3 serum^16^, rabbit anti-DHAV-1 serum and normal rabbit serum used in this experiment were all provided by the Sichuan Agricultural University Poultry Disease Prevention Research Center. A rabbit anti-FLAG polyclonal antibody and a rabbit anti-human β-actin polyclonal antibody were purchased from Shanghai Biyuntian Biotechnology Co., Ltd.

### Plasmids

pGEX-4T-1/VP3^16^ and the eukaryotic expression vector pCAGGS were provided by the Sichuan Agricultural University Poultry Disease Prevention Research Center. The primers and probes used in the experiment were synthesized by Shanghai Keikang Biotechnology Co., Ltd. The primers used are shown in Table 1.

**Table 1:** Sequences of the primers used in this study.

Gene	Forward primer	Reverse primer
VP0	5′-CCATCTGTGTCATTGTGTTAGGCA-3′	5′-CAAATCAGTTTCAAGGAGTTCTCCA-3′
Caspase-3	5′-TGGTGTTGAGGCAGACAGTGGA-3′	5′-CATTCCGCCAGGAGTAATAGCC-3′
Caspase-8	5′-GGTGATGCTCGTCAGAAAGGTG-3′	5′-AGCCATGCCCAAGAGGAAGT-3′
Caspase-9	5′-GCTGCTTCAACTTCCTCCGTAA-3′	5′-CATCTCCACGGACAGACAAAGG-3′
β-Actin	5′-CCGGGCATCGCTGACA-3′	5′-GGATTCATCATACTCCTGCTTTGCT-3′
VP3	5′-CATCATTTTGGCAAAGAATTCGCCAC CATGGGAAAGAGAAAACCATGCAGG-3′	5′-TTGGCAGAGGGAAAAAGATCTTCAC TTATCGTCGTCATCCTTGTAATCTTGATTGTTAGTTGCCATCTGC-3′

Note: The VP3 forward primer included the 15 bp upstream vector terminal homologous sequence (indicated by the solid line below) and an EcoRI restriction site (shown in the box). The 5′ terminus of the reverse primer included the 15 bp downstream vector terminal homologous sequence (shown under the solid line), a Bgl II cleavage site (shown in the box) and a FLAG tag (indicated by the wavy line).

### RNA isolation and cDNA preparation

Total RNA was isolated from the samples using the RNAiso Plus Reagent (TaKaRa) according to the instructions and reverse transcribed using the PrimeScriptTM RT kit (Perfect Real Time, TaKaRa) according to the instructions.

### qRT-PCR

Viral copy number was determined by a one-step TaqMan probe real-time fluorescent quantitative RT-PCR method^27,50^.

### Expression of VP3 protein after transformation of pGEX-4T-1/VP3 prokaryotic plasmid into *Escherichia coli* BL21

The pGEX-4T-1/VP3 recombinant plasmid was extracted according to the protocol of a plasmid extraction kit (Omega), and the recombinant plasmid was transformed into the *E*. *coli* BL21 (DE3) expression host strain by the CaCl_2_ method. The transformed strain was cultured for 8 h at an induction temperature of 30 °C and a final concentration of 0.2 mmol/L IPTG. After lysing the bacteria, the supernatant was collected, and the VP3 protein was purified as described^24^ and stored at −20 °C.

### Construction and transfection of the pCAGGS/VP3 eukaryotic expression plasmid

The specific sequence of the capsid protein VP3 was amplified by cDNA PCR using specific primers (Table 1). The VP3 fragment was ligated with the linearized pCAGGS fragment according to previous work^51^ and transferred into competent *E*. *coli* DH5α cells; suspected positive single colonies were selected for PCR identification and restriction enzyme digestion. The positive clones were sent to Invitrogen for sequencing, and the confirmed plasmid was named pCAGGS/VP3. The pCAGGS/VP3 plasmid was transferred into duck embryo fibroblasts (DEFs) according to the instructions for the Lipofectamine® 3000 (Invitrogen) transfection reagent^45^. The dose of DHAV-1 was 0.1 MOI, and DHAV-1 was added to DEF monolayer cells for 1 h at 37 °C. The cells were cultured in MEM supplemented with 2% NBS.

### Western blotting and indirect immunofluorescence assay (IFA)

Cell samples were collected after pCAGGS/VP3 transfection for 48 h; rabbit anti-VP3 serum (1:100) was used as the primary antibody, and HRP-labeled goat anti-rabbit IgG (1:3000) was used as the secondary antibody. Western blotting was performed according to previous work^51^. DEFs transfected with pCAGGS/VP3 for 48 h and DEFs infected with DHAV-1 for 48 h were fixed with 4% paraformaldehyde, permeabilized with 0.25% Triton in PBS, and blocked with 5% BSA in PBS. Rabbit anti-VP3 serum (1:100) and Alexa Fluor 488-labeled goat anti-rabbit IgG (1:1000) were used as primary and secondary antibodies, respectively. Nuclei were stained using DAPI, and the cell slides were blocked with glycerol. The results were observed using an inverted fluorescence microscope.

### DAPI staining, TUNEL staining and flow cytometry

pCAGGS/VP3 and pCAGGS liposomes were transfected into DEFs, and cell slides were prepared at different times. The cells were fixed, permeabilized, and incubated with DAPI at 37 °C for 15 min in the dark; the cell slides were blocked with glycerol. Apoptosis Detection Kit (Buddhist) was used for staining according to the instructions, followed by blocking with glycerol; the slides were observed under an inverted fluorescence microscope. After transfecting pCAGGS/VP3 and pCAGGS liposomes into DEFs for 48 h, 3 repetitions for each transfection, the cells were stained according to the apoptotic double staining assay kit (BD). The samples were sent to a company for flow cytometry. The sum of the proportions of cells stained with only Annexin V-FITC and those simultaneously stained with Annexin V-FITC and PI was used as the total proportion of apoptotic cells. T test analysis was used to assess significant differences between the experimental group (pCAGGS/VP3) and the control group (pCAGGS).

### Detection of apoptotic transcriptional levels and detection of caspase-3, −8 and −9 enzyme activities

pCAGGS/VP3 and pCAGGS were transfected into DEFs. After transfection for 48 h, cell samples were collected for reverse transcription. Primers for apoptotic proteins, including caspase-3, caspase-8, caspase-9, and the internal reference gene β-actin were designed according to the design principles for real-time fluorescent quantitative PCR primers and sequences published in GenBank (Table 1). The reaction system and reaction conditions were determined according to the instructions of SYBR® Premix Ex Taq^TM^ II (TaKaRa) to analyze the transcriptional levels of caspase-3, −8 and −9 in DEFs after overexpression of VP3; the same method was applied for detecting the transcriptional levels of other apoptotic factors. DEFs were transfected with pCAGGS/VP3 or pCAGGS for 48 h. In a 96-well plate, 100 μL of a cell (1~5 × 104 cells) suspension and 100 μL of caspase-3, caspase-8 and caspase-9 enzyme activity detection reagent (Promega) was added separately and mixed; the cells were incubated at 22 °C for 1 h in the dark and analyzed using a multifunction microplate reader within 3 h.

### Detection of mitochondrial membrane potential

pCAGGS-VP3 was transfected into DEF monolayer cells, and the mitochondrial membrane potential of DEFs was measured according to the JC-1 staining kit (Sigma-Aldrich) manual at 12 h, 24 h, 36 h, 48 h, 60 h, and 72 h after transfection. JC-1 dye was added to DEF monolayer cells during the assay and incubated at 37 °C for 20 min in the dark. Fluorescence was detected using a multi-function microplate reader.

## Supplementary information

Supplementary Figures and Figure Legends

LaTeX Supplementary File

LaTeX Supplementary File

## References

[1] Yugo DM, Hauck R, Shivaprasad HL, Meng XJ (2016). Hepatitis Virus Infections in Poultry. Avian diseases.

[2] Haider SA, Calnek BW (1979). *In vitro* isolation, propagation, and characterization of duck hepatitis virus type III. Avian diseases.

[3] Toth TE (1969). Studies of an agent causing mortality among ducklings immune to duck virus hepatitis. Avian diseases.

[4] Todd D (2009). Identification of chicken enterovirus-like viruses, duck hepatitis virus type 2 and duck hepatitis virus type 3 as astroviruses. Avian pathology: journal of the W.V.P.A.

[5] Wang L, Pan M, Fu Y, Zhang D (2008). Classification of duck hepatitis virus into three genotypes based on molecular evolutionary analysis. Virus genes.

[6] Tseng CH, Tsai HJ (2007). Molecular characterization of a new serotype of duck hepatitis virus. Virus research.

[7] Kim MC (2007). Recent Korean isolates of duck hepatitis virus reveal the presence of a new geno- and serotype when compared to duck hepatitis virus type 1 type strains. Archives of virology.

[8] Fu Y (2008). Molecular detection and typing of duck hepatitis A virus directly from clinical specimens. Veterinary microbiology.

[9] Wen X (2018). Molecular epidemiology of duck hepatitis a virus types 1 and 3 in China, 2010–2015. Transboundary and emerging diseases.

[10] Kim MC (2006). Molecular analysis of duck hepatitis virus type 1 reveals a novel lineage close to the genus Parechovirus in the family Picornaviridae. The Journal of general virology.

[11] Sun D (2017). Cleavage of poly(A)-binding protein by duck hepatitis A virus 3C protease. Scientific reports.

[12] Yang X (2017). Structures and Corresponding Functions of Five Types of Picornaviral 2A Proteins. Frontiers in microbiology.

[13] Wen X (2015). Recent advances from studies on the role of structural proteins in enterovirus infection. Future microbiology.

[14] Zhang Y (2015). [Construction and characterization of an epitope-mutated Asia 1 type foot-and-mouth disease virus]. Sheng wu gong cheng xue bao = Chinese journal of biotechnology.

[15] Jian-Sheng, X. U. *et al*. Construction and Immunization of Recombinants of the Structural Protein VP3 Gene of Avian Encephalomyelitis Virus. *Chinese Journal of Veterinary Science* (2006).

[16] Shen, Y. L. *et al*. Neutralizing Activity Analysis of VP3 Antiserums and B-cell Epitopes Identification of VP3 Protein form Duck Hepatitis A Virus Type 1. *Chinese Journal of Animal & Veterinary Sciences* (2016).

[17] Yauch RL, Kerekes K, Saujani K, Kim BS (1995). Identification of a major T-cell epitope within VP3 amino acid residues 24 to 37 of Theiler’s virus in demyelination-susceptible SJL/J mice. Journal of virology.

[18] Bong-Su K, Lyman MA, Kim BS (2002). The majority of infiltrating CD8+ T cells in the central nervous system of susceptible SJL/J mice infected with Theiler’s virus are virus specific and fully functional. Journal of virology.

[19] Morrell DJ, Mellor EJ, Rowlands DJ, Brown F (1987). Surface structure and RNA-protein interactions of foot-and-mouth disease virus. The Journal of general virology.

[20] Liu J, Wei T, Kwang J (2002). Avian encephalomyelitis virus induces apoptosis via major structural protein VP3. Virology.

[21] Xie J (2018). Transcriptomic Characterization of a Chicken Embryo Model Infected With Duck Hepatitis A Virus Type 1. Frontiers in immunology.

[22] Xie J (2018). Cytokine storms are primarily responsible for the rapid death of ducklings infected with duck hepatitis A virus type 1. Scientific reports.

[23] Mao S (2017). Virologic and Immunologic Characteristics in Mature Ducks with Acute Duck Hepatitis A Virus 1. Infection. Frontiers in immunology.

[24] Ou X (2017). The neglected avian hepatotropic virus induces acute and chronic hepatitis in ducks: an alternative model for hepatology. Oncotarget.

[25] Ou X (2017). Viral-host interaction in kidney reveals strategies to escape host immunity and persistently shed virus to the urine. Oncotarget.

[26] Mao S (2016). Development and evaluation of indirect ELISAs for the detection of IgG, IgM and IgA1 against duck hepatitis A virus 1. Journal of virological methods.

[27] Hu Q (2016). A one-step duplex rRT-PCR assay for the simultaneous detection of duck hepatitis A virus genotypes 1 and 3. Journal of virological methods.

[28] Wen XJ (2014). Detection, differentiation, and VP1 sequencing of duck hepatitis A virus type 1 and type 3 by a 1-step duplex reverse-transcription PCR assay. Poultry science.

[29] Anchun C (2009). Development and application of a reverse transcriptase polymerase chain reaction to detect Chinese isolates of duck hepatitis virus type 1. Journal of microbiological methods.

[30] Ou X (2018). Incompatible Translation Drives a Convergent Evolution and Viral Attenuation During the Development of Live Attenuated. Vaccine. Frontiers in cellular and infection microbiology.

[31] Zhang Y (2017). The 3D protein of duck hepatitis A virus type 1 binds to a viral genomic 3′ UTR and shows RNA-dependent RNA polymerase activity. Virus genes.

[32] Sun D, Chen S, Cheng A, Wang M (2016). Roles of the Picornaviral 3C Proteinase in the Viral Life Cycle and Host Cells. Viruses.

[33] Levine PP, Fabricant J (1950). A hitherto-undescribed virus disease of ducks in North America. Cornell Veterinarian.

[34] Song C (2014). Effect of age on the pathogenesis of DHV-1 in Pekin ducks and on the innate immune responses of ducks to infection. Archives of virology.

[35] Ou X (2017). Comparative analysis of virus-host interactions caused by a virulent and an attenuated duck hepatitis A virus genotype 1. PloS one.

[36] Bergelson JM, Coyne CB (2013). Picornavirus entry. Advances in experimental medicine and biology.

[37] Smyth MS, Martin JH (2002). Picornavirus uncoating. Molecular pathology: MP.

[38] Jackson T (1996). Efficient infection of cells in culture by type O foot-and-mouth disease virus requires binding to cell surface heparan sulfate. Journal of virology.

[39] Anil KU (2012). Sequence analysis of capsid coding region of foot-and-mouth disease virus type A vaccine strain during serial passages in BHK-21 adherent and suspension cells. Biologicals: journal of the International Association of Biological Standardization.

[40] Fry EE (1999). The structure and function of a foot-and-mouth disease virus-oligosaccharide receptor complex. The EMBO journal.

[41] Sa-Carvalho D (1997). Tissue culture adaptation of foot-and-mouth disease virus selects viruses that bind to heparin and are attenuated in cattle. Journal of virology.

[42] Li X (2017). Evidence of VP1 of duck hepatitis A type 1 virus as a target of neutralizing antibodies and involving receptor-binding activity. Virus research.

[43] Peng JM, Liang SM, Liang CM (2004). VP1 of foot-and-mouth disease virus induces apoptosis via the Akt signaling pathway. The Journal of biological chemistry.

[44] Henke A (2000). Apoptosis in coxsackievirus B3-caused diseases: interaction between the capsid protein VP2 and the proapoptotic protein siva. Journal of virology.

[45] Cao J (2016). The 2A2 protein of Duck hepatitis A virus type 1 induces apoptosis in primary cell culture. Virus genes.

[46] Buenz EJ, Howe CL (2006). Picornaviruses and cell death. Trends in microbiology.

[47] Clarke P, Tyler KL (2009). Apoptosis in animal models of virus-induced disease. Nature reviews. Microbiology.

[48] Kroemer G, Reed JC (2000). Mitochondrial control of cell death. Nat Med.

[49] Wang G (2013). Small-molecule activation of the TRAIL receptor DR5 in human cancer cells. Nature Chemical Biology.

[50] Yang M, Cheng A, Wang M, Xing H (2008). Development and application of a one-step real-time Taqman RT-PCR assay for detection of Duck hepatitis virus type1. Journal of virological methods.

[51] Joseph GMRS (2000). Molecular Cloning: A Laboratory Manual, Fourth Edition 3 vol.set. Analytical Biochemistry.

[52] Karupiah G (2002). Fields Virology, 4th Edition. Immunology & Cell Biology.

[53] Garmaroudi FS (2015). Coxsackievirus B3 replication and pathogenesis. Future microbiology.

[54] Baggen J, Thibaut HJ, Strating J, van Kuppeveld FJM (2018). The life cycle of non-polio enteroviruses and how to target it. Nature reviews. Microbiology.

