# Supplementary material for: The VP3 protein of duck hepatitis A virus mediates host cell adsorption and apoptosis
Source: Sci Rep. 2019 Nov 14;9:16783. doi: 10.1038/s41598-019-53285-0 (PMC6856352; doi:10.1038/s41598-019-53285-0)
Supplement: Supplementary file 1 — Supplementary Figures and Figure Legends [file 41598_2019_53285_MOESM1_ESM.docx]

VP3 protein of duck hepatitis A virus mediates host cell adsorption and apoptosis

Yalan Lai^1,2,3^, Zeng Ni^1,2,3#^, MingshuWang^1,2,3#*^, Anchun Cheng^1,2,3*^, Qiao Yang^1,2,3^, Ying Wu^1,2,3^, Renyong Jia^1,2,3^, Dekang Zhu^2,3^, XinXin Zhao^1,2,3^, Shun Chen^1,2,3^, Mafeng Liu^1,2,3^, Shaqiu Zhang^1,2,3^, Yin Wang^2^, Zhiwen Xu^2^, Zhengli Chen^2^, Ling zhu^2^, Qihui Luo^2^, Yunya Liu^1,2,3^, Yanling Yu^1,2,3^, Ling Zhang^1,2,3^, Huang Juan^1,2,3^, Bin Tian^1,3^, Leichang Pan^1,3^, Mujeeb Ur Rehman^1,3^, Xiaoyue Chen^2,3^

**Supplementary Figures and Figure Legends**


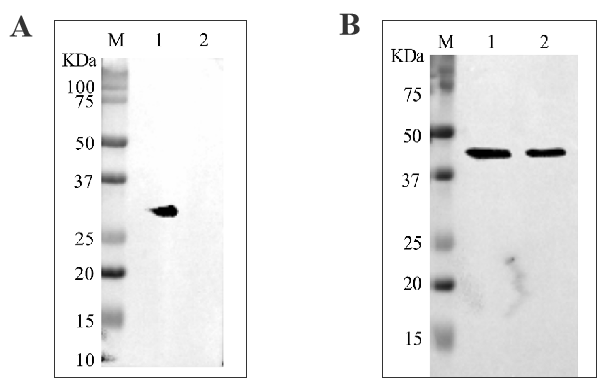


Figure 11. Expression of eukaryotic recombinant plasmid pCAGGS/VP3. Lane M is 10 KDa-250 KDa protein Marker. (A) Expression of VP3 after transfection of pCAGGS/VP3 (Lane 1) and pCAGGS (Lane 2) into DEFs. The VP3 protein is approximately 28 kDa. (B) Expression of β-actin after transfection of pCAGGS/VP3 (Lane 1) and pCAGGS (Lane 2) into DEFs. The β-actin protein is approximately 43 kDa.


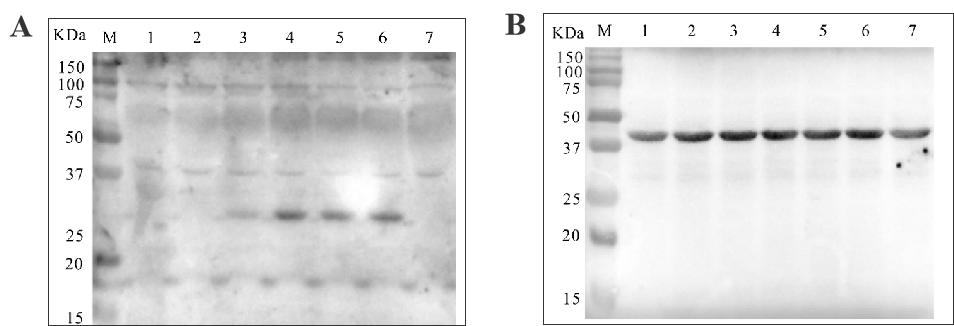


Figure 12. Expression of VP3 protein after DHAV-1 infection of DEFs. Lane M is 10 KDa-250 KDa protein Marker. Lanes 1-6 are samples of DEFs infected with DHAV-1 for 12 h, 24 h, 36 h, 48 h, 72 h, and 84 h, respectively. Lane 7 is a sample of DEF that is not infected with DHAV-1. (A) Expression of VP3 protein after DHAV-1 infection of DEFs. The VP3 protein is approximately 28 kDa. (B) Expression of β-actin after DHAV-1 infection of DEFs. The β-actin protein is approximately 43 kDa.
